# Crystal Structures of Epstein–Barr Virus Bcl-2 Homolog BHRF1 Bound to Bid and Puma BH3 Motif Peptides

**DOI:** 10.3390/v14102222

**Published:** 2022-10-09

**Authors:** Chathura D. Suraweera, Mark G. Hinds, Marc Kvansakul

**Affiliations:** 1 Department of Biochemistry & Chemistry, La Trobe Institute for Molecular Science, La Trobe University, Melbourne, VIC 3086, Australia; 2 Bio21 Molecular Science and Biotechnology Institute, The University of Melbourne, Parkville, VIC 3010, Australia

**Keywords:** Epstein–Barr virus, BHRF1, Bcl-2, apoptosis, crystallography

## Abstract

Apoptosis is a powerful defense mechanism used by multicellular organisms to counteract viral infection. In response to premature host cell suicide, viruses have evolved numerous countermeasures to ensure cell viability to optimize their replication by encoding proteins homologous in structure and function to cellular pro-survival Bcl-2 proteins. Epstein–Barr virus (EBV), a member of the *Gammaherpesviridae*, encodes the Bcl-2 homolog BHRF1, a potent inhibitor of Bcl-2-mediated apoptosis. BHRF1 acts by directly targeting Bid and Puma, two proapoptotic proteins of the Bcl-2 family. Here, we determined the crystal structures of BHRF1 bound to peptides spanning the Bcl-2 binding motifs (Bcl-2 homology 3 motif, BH3) of Bid and Puma. BHRF1 engages BH3 peptides using the canonical ligand-binding groove of its Bcl-2 fold and maintains a salt bridge between an Arg residue with a conserved Asp residue in the BH3 motif mimicking the canonical ionic interaction seen in host Bcl-2:BH3 motif complexes. Furthermore, both Bid and Puma utilize a fifth binding pocket in the canonical ligand binding groove of BHRF1 to provide an additional hydrophobic interaction distinct from the interactions previously seen with Bak and Bim. These findings provide a structural basis for EBV-mediated suppression of host cell apoptosis and reveal the flexibility of virus encoded Bcl-2 proteins in mimicking key interactions from the endogenous host signaling pathways.

## 1. Introduction

Epstein–Barr virus (EBV) is a member of the *Gammaherpesviridae* genus and is the causative agent of infectious mononucleosis (glandular fever) in humans and implicated in lymphoproliferative disorders, gastric, and nasopharyngeal cancer [1]. EBV preferentially infects B-lymphocytes, and it is estimated that greater than 90% of the world adult population has a latent asymptomatic EBV infection [2]. Among the lymphoproliferative diseases associated with EBV are Burkitt’s lymphoma, a quick-onset pediatric B lymphoid-associated carcinoma frequently found in sub-Saharan Africa [3], and Hodgkin’s lymphoma [4]. EBV infection has been closely associated with the development of the autoimmune disease multiple sclerosis (MS) [5,6]. The underlying mechanisms of these diseases is the expression of viral proteins that function to manipulate the host-cell innate immune response and, in particular, inhibit host-cell apoptosis.

Mitochondrial-mediated or intrinsic apoptosis is regulated by the B-cell lymphoma 2 (Bcl-2) family proteins. Activation of proapoptotic members of the Bcl-2 family leads to mitochondrial outer membrane permeabilization (MOMP) and release of cytochrome *c* and other factors into the cytoplasm that activate the cell-destroying caspase cascade [7,8,9]. The proapoptotic Bcl-2 members are antagonized by their interactions with their pro-survival Bcl-2 counterparts [9]. The mammalian pro-survival Bcl-2 proteins comprise Bcl-2, Bcl-x_L_, Bcl-w, Mcl-1, A1/Bfl1, and Bcl-B, which function to inhibit intrinsic apoptosis [8]. In contrast, proapoptotic Bcl-2 proteins categorized into two groups: multi-motif apoptosis effectors (Bak, Bax, Bok, and Bid) and BH3-only proteins (Bad, Bid, Bik, Bim, Bmf, Hrk, Noxa, and Puma) [8,10]. The expression levels and interaction between the antagonistic pro-survival and proapoptotic Bcl-2 proteins determines cellular fate [8,9]. The critical role of the Bcl-2 family in life and death decisions in the cell has led to their mimicry by viruses [11].

Numerous large double-stranded DNA viruses, including examples from *Poxviridae*, *Herpesviridae*, *Iridoviridae*, *Adenoviridae*, and *Asfraviridae*, have evolved sophisticated immunomodulatory strategies to counter host-cell innate and adaptive immune responses to viral infection. One such strategy is to block host-cell intracellular responses to infection such as apoptosis to viral proliferation, allowing infection to proceed [12,13,14]. This includes expression of direct inhibitors against caspases and mimicry of cellular Bcl-2 proteins to inactivate host proapoptotic proteins and block host-cell intrinsic apoptosis [14]. Viral Bcl-2 (vBcl-2) proteins are highly sequence divergent, but structurally and functionally are well-conserved homologs of cellular pro-survival Bcl-2 proteins [11,13]. Examples from *Herpesviridae* include those from murine gamma herpesvirus M11 [15], Karposi sarcoma herpes virus (KSHV) KsBcl-2 [16,17], and Epstein–Barr virus (EBV), which unusually bears two Bcl-2 homologs BHRF1 and BALF1 [18,19].

The genome of EBV encodes BHRF1, and elevated expression of BHRF1 leads to resistance to multiple cytotoxic agents [18,20]. During such overexpression of BHRF1, mouse progenitor cells and hematopoietic stem cells have an increased chance of MYC-induced lymphoma development in a mouse model [18]. EBV BHRF1 is a Bcl-2 homolog that potently inhibits host intrinsic apoptosis via direct inactivation of cellular proapoptotic Bcl-2 proteins Bak and Bim [21,22] and selectively interacts with a subset of proapoptotic proteins (Bid, Puma, and Bax) [23] to regulate apoptosis. Here, we report the structural investigation of BHRF1 bound to peptides that span the Bid BH3 and Puma BH3 motif. Our findings provide structural and mechanistic insight into EBV BHRF1-mediated inhibition of apoptosis.

## 2. Materials and Methods

### 2.1. Protein Expression and Purification

A synthetic cDNA codon-optimized for *Escherichia coli* encoding the construct EBV BHRF1ΔC31 (EBV strain B95-8, spanning residues 1–160 with a C-terminal deletion of 31 residues [18]; UniProt KB P03182 and referred to hereafter as BHRF1) was cloned into the bacterial expression vector pET-Duet1 (Genscript) and transformed into *E. coli* Codon plus (RIL). Protein expression and purification were carried out as previously described [18]. Briefly, BHRF1-expressing *E. coli* cells were resuspended in 50 mL of lysis buffer A (50 mM Tris, pH 8.5, 300 mM NaCl, and 20 mM imidazole). The cells were lysed using sonication (program 6 (6 s pulse-on, 60 s pulse-off) Model 705 Sonic Dismembrator, Fisher Scientific, Hampton, NH, USA), and the resultant lysate was transferred into SS34 tubes for further centrifugation at 20,000 rpm (JA-25.50 rotor, Beckman Coulter Avanti J-E, Beckman Coulter Australia) for 30 min. The supernatant was loaded onto a HisTrap HP column, 5 mL (GE Healthcare, Little Chalford, UK), equilibrated with buffer A. After sample application, the column was washed with 100 mL of buffer A, and the target protein was eluted with buffer B (50 mM Tris, pH 8.5, 300 mM NaCl, and 300 mM imidazole) and concentrated to a final volume of 5 mL using a centrifugal concentrator with a 3 kDa molecular weight cutoff (Amicon^®^ Ultra 15, Merck Pty. Ltd., Melbourne, Australia). Concentrated BHRF1 protein was subjected to size-exclusion chromatography using a Superdex S75 16/60 column mounted on an ÄKTAExpress system (GE Healthcare) equilibrated in 25 mM HEPES pH 7.5, 150 mM NaCl, 5 mM TCEP (Tris(2-carboxyethyl)phosphine hydrochloride), where it eluted as a single peak in the volume corresponding to the monomeric nature. The final sample purity was estimated to be higher than 95% according to SDS-PAGE analysis.

### 2.2. Crystallization and Structure Determination

Crystals for BHRF1–BidBH3 and BHRF1–PumaBH3 complexes were obtained by mixing BHRF1 with human Bid (34-mer, 76-SESQEDIIRNIARHLAQVGDSMDRSIPPGLVNGL-109): Puma (26-mer, 130-EEQWAREIGAQLRRMADDLNAQYERR-155) peptide using a 1:1.5 molar ratio as described [24] and concentrated using a centrifugal concentrator with 3 kDa molecular weight cutoff (Amicon^®^ Ultra 0.5, Merck Pty. Ltd., Melbourne, Australia) to 5 mg/mL. Concentrated protein was immediately used for crystallization trials. Initial high-throughput sparse matrix screening was performed using 96-well sitting drop trays (Swissic, Neuheim, Switzerland).

BHRF1–BidBH3 crystals were grown using the sitting drop vapor diffusion method at 20 °C in 0.1 M sodium acetate, pH 4.6, 8% (*w*/*v*) PEG 4000, or 0.4 M ammonium phosphate monobasic. The crystals were flash-cooled at −173 °C in mother liquor supplemented with 20% ethylene glycol. The BHRF1–BidBH3 complex formed single cuboidal crystals belonging to space group P6_5_22 with a = 94.20 Å, b = 94.20 Å, c = 455.58 Å, α = 90.0°, *β* = 90.0°, and *γ* = 120.0° in the hexagonal crystal system.

Diffraction data were acquired at the Australian Synchrotron MX2 beamline [25] using an Eiger 16M detector with an oscillation range of 0.1° per frame and a wavelength of 0.9537 Å. The diffraction data were integrated using XDS [26] and scaled using AIMLESS [27]. A molecular replacement solution was obtained using PHASER as implemented in PHENIX [28], using the previously solved structure of BHRF1–BimBH3 (PDB ID 2WH6) [18] as the initial search model. BHRF1–BidBH3 crystals contained five molecules of BHRF1 and five BidBH3 peptides in the asymmetric unit, with a 53.7% solvent content and final TFZ and LLG values of 47.3 and 3316.2, respectively. The final model of the BHRF1–BidBH3 complex was built manually over several cycles using Coot [29] and refined using PHENIX [30] with final R_work_/R_free_ values of 0.231/0.274, yielding a model where 99% of residues were in the favored region of the Ramachandran plot with no outliers.

BHRF1–PumaBH3 crystals were obtained in 0.4 M ammonium monobasic phosphate. The crystals were flash-cooled at −173 °C in mother liquor supplemented with 20% ethylene glycol as cryoprotectant. The complex formed single cubic crystals belonging to space group P3_2_21 with a = 62.77 Å, b = 62.77 Å, c = 92.60 Å, α = 90.00°, *β* = 90.00°, and *γ* = 120.00° in the trigonal crystal system. Diffraction data collection, integration, and scaling were performed as described above. The molecular replacement was carried out using PHASER [28] as described above. BHRF1–PumaBH3 crystals contained a single molecule of BHRF1 and a single PumaBH3 peptide, with a 47.93% solvent content and final TFZ and LLG values of 34.5 and 4338.35, respectively. The final model of BHRF1–PumaBH3 was built manually over several cycles using Coot [29] and refined using PHENIX [30] with final R_work_/R_free_ values of 21.7/25.2, yielding a model where 99% of residues were in the favored region of the Ramachandran plot with no outliers.

The X-ray crystallographic data collection and refinement statistics for the structures determined are presented in Table 1. Sequence and structural analysis was performed using the DALI server [31], and sequence statistics are presented in Table 2. In the absence of an experimental structure of F72W BHRF1, AlphaFold (version 2.1.0) was used to model the BHRF1 mutant that selectively binds Puma [20], using the Google Colab online server. The structure of F72W BHRF1(1–160) was calculated by providing the sequence with the standard parameters provided [32]. The model produced was built with the per-residue accuracy of the structure as determined by the predicted local distance difference test (pLDDT) at the >90% confidence level over the entire sequence, and it was further confirmed by superimposition with experimentally determined structure of BHRF1–Puma.

All images for BHRF1–BidBH3 and BHRF1–PumaBH3 complexes and superimpositions were generated using PyMOL molecular graphic system version 1.8.6.0 (Schrödinger, LLC, New York, NY, USA). The two structures were deposited at the Protein Data Bank with PDB accession codes 7P33 and 7P9W for the BidBH3 and PumaBH3 complexes, respectively. All raw images were deposited at the SBGridDB [33] using their PDB accession codes. All software was accessed through the SBGrid suite [34].

## 3. Results

**Figure 1:**
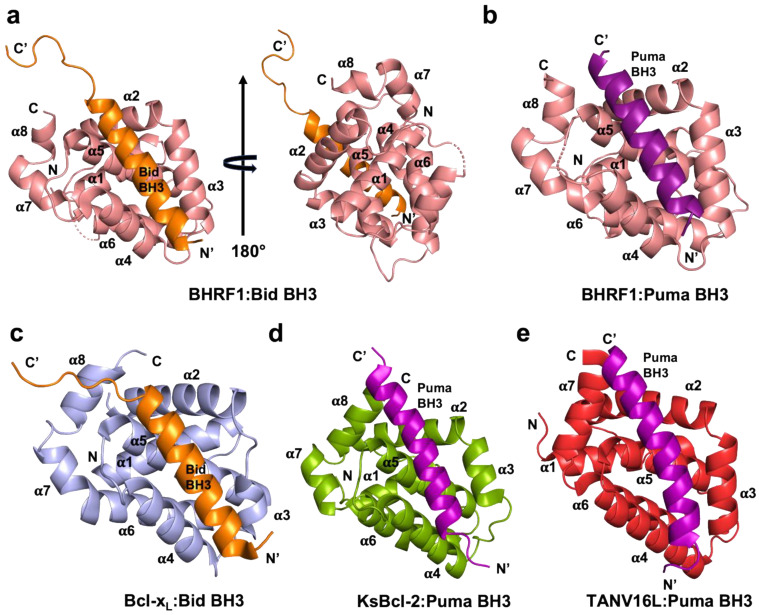
EBV BHRF1 binds BH3 motif peptides of proapoptotic Bcl-2 proteins using the canonical ligand-binding groove. (**a**) Ribbon representation of the crystal structure of BHRF1–Bid BH3 (PDB ID 7P33) complex and its 180° rotation. BHRF1 is shown in salmon, while BidBH3 is shown in orange, with BHRF1 helices labeled α1–α8. The view is into the hydrophobic binding groove formed by helices α2–α5. (**b**) Ribbon representation of the crystal structure of BHRF1–Puma BH3 (PDB ID 7P9W) complex. BHRF1 is shown as the salmon cartoon, while PumaBH3 is shown in purple. The view is as in (**a**). (**c**) Cartoon of Bcl-x_L_–BidBH3 complex. Human Bcl-x_L_ is shown in light blue, while Bid is shown in orange. (**d**) Cartoon of KSHV KsBcl-2–Puma BH3 complex. KsBcl-2 is shown as an olive-colored cartoon, while Puma is shown in purple. I Cartoon of tanapoxvirus TANV16L–Puma BH3 complex. TANV16L is shown as a red-colored cartoon, while Puma is shown in purple. The views in (**c**–**e**) are as in (**a**).

**Figure 2:**
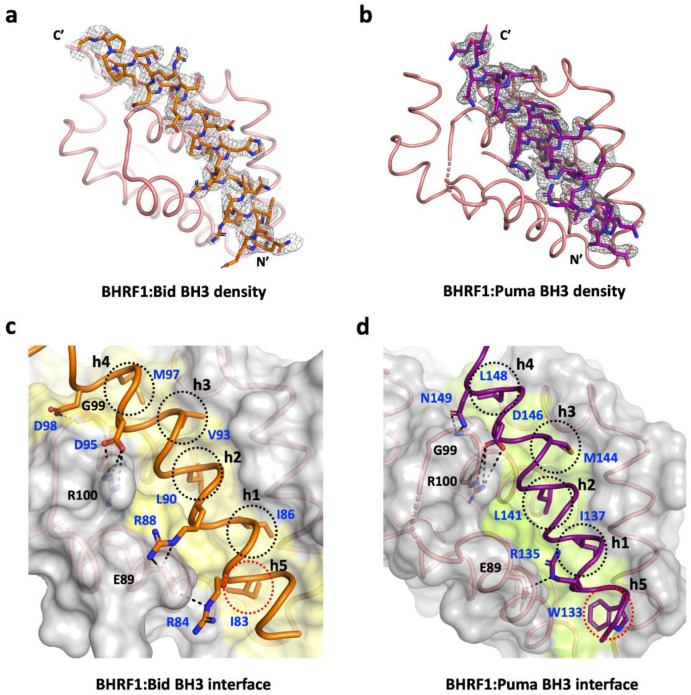
Electron density maps of BHRF1–Bid BH3 and Puma BH3 complexes and detailed view of the BHRF1–Bid BH3 and Puma BH3 interfaces. (**a**) The 2Fo-Fc electron density maps of BHRF1–Bid BH3 complex interface contoured at 1.0 σ. BHRF1 is shown as salmon ribbon, while Bid BH3 is shown as orange-colored sticks. (**b**) The 2Fo-Fc electron density maps of BHRF1–PumaBH3 complex interface contoured at 1.0 σ. BHRF1 is shown as a salmon ribbon, while Puma BH3 is shown as purple-colored sticks. Electron density distribution is shown in gray. (**c**) The BHRF1 backbone, floor of the binding groove, and surface are shown in salmon, yellow, and gray, respectively, whilst the Bid BH3 ribbon is shown in orange. The five hydrophobic residues of Bid BH3 (I83, I86, L90, V93, and M97) are protruding into the binding groove, and conserved ionic interactions formed by BHRF1 R100 and Bid BH3 D95 are labeled, as well as all other residues involved in ionic interactions and hydrogen bonds between protein and peptide. (**d**) BHRF1 is shown as in (**c**), whilst the Puma BH3 ribbon is shown in purple. The five hydrophobic residues of Puma BH3 (W133, I137, L141 M144, and L148) are protruding into the binding groove, and the conserved salt bridge interaction formed by BHRF1 R100 and Puma BH3 D146 is labeled, as well as other residues involved in additional ionic interactions and hydrogen bonds. Ionic interactions and hydrogen bonds are shown as dotted black lines, and the participating residues are labeled. The canonical hydrophobic binding pockets h1–h4 are indicated by black circles, while pocket h5 is indicated by a red circle. The molecular orientations depicted in (**a**–**d**) are identical to those in Figure 1.

It was previously reported that the EBV Bcl-2 homolog BHRF1 has a restricted BH3 interaction profile toward peptides spanning the BH3 motif (26-mer) of proapoptotic Bcl-2 proteins and binds only human Bak, Bax, Bim, Bid, and Puma BH3-motif peptides with nanomolar to sub-micromolar affinities [18]. To understand the molecular basis of the interaction between BHRF1 and Bid BH3 and Puma BH3 in modulating the inhibition of apoptosis, we determined the crystal structures of BHRF1 in complex with BidBH3 (Figure 1a) and PumaBH3 (Figure 1b). The structure of the BHRF1–BidBH3 complex was refined to a resolution of 2.78 Å, whereas the BHRF1–Puma BH3 complex was refined to 2.0 Å (Table 1) resolution. Both crystal structures of BHRF1 BH3 complexes were solved by molecular replacement using the previously solved structure of BHRF1–BimBH3 (PDB ID 2WH6) [18] as an initial search model. For the BHRF1–Bid BH3 complex, a clear and continuous electron density map was observed for BHRF1 residues 3–155 and BidBH3 motif residues 78–107 (Figure 2a). Despite sharing very weak sequence identity with its cellular counterparts Bcl-2 (21.7%), Bcl-x_L_ (17.9%), Bcl-w (18.4%), Mcl-1 (9.6%), Bfl-1/A1 (17.8%), and Bcl-B (21.5%), BHRF1 adopts the conserved alpha helical Bcl-2 fold comprising eight α-helices (Figure 1a,b), with helices α2–α5 forming the canonical hydrophobic ligand-binding groove which provides the interaction site for the BH3-motif peptide (Figure 1a,b). The crystal structure of BHRF1 revealed a globular monomeric configuration that has been observed in other Bcl-2 homologs including human Bcl-x_L_ (Figure 1c) and herpesvirus Bcl-2 homologs including KSHV KsBcl-2 (Figure 1d) [17], murine gamma herpes virus Bcl-2 homolog M11 [15], several poxviruses including sheep poxvirus SPPV14 [35,36], myxomavirus M11L [37], tanapoxvirus TANV16L [38] (Figure 1e) (which adopts both monomeric and dimeric configurations), fowl poxvirus FPV039 [39], canary poxvirus CNP058 [40], and African swine fever virus (ASFV) A179L [41,42]. A structural investigation and similarity analysis was performed using DALI server [31], which identified KSHV KsBcl-2 (PDB ID 1K3K, 7QTX) [17] as the closest viral Bcl-2 homolog of the BHRF1–Bid and BHRF1–Puma complexes with an RMSD of 2.8 Å over 116 Cα atoms and human Bcl-x_L_ (PDB ID 2M04) (Figure 1c) [43] as the closest cellular Bcl-2 homolog with an RMSD of 2.5 Å over 133 Cα atoms where there was no significant difference in RMSD value observed for searches of the BH3-motif complexes. Bid BH3 is bound to BHRF1 via the canonical hydrophobic ligand-binding groove (Figure 1a). The four canonical BH3 motif-defining residues from Bid, I86, L90, V93, and M97, engage four hydrophobic pockets of the canonical BHRF1 ligand-binding groove with an additional hydrophobic pocket occupied by a fifth hydrophobic residue, I83 (Figure 2c). Furthermore, these hydrophobic interactions are supplemented with two ionic interactions (salt bridges) between D95^Bid^ and R100^BHRF1^, the hallmark of the pro-survival Bcl-2 interaction between a conserved arginine in the BH1 motif and aspartate of the BH3 motif, which is found in almost all pro-survival Bcl-2–BH3 complexes [9] and R88^Bid^ and E89^BHRF1^, as well as two hydrogen bonding interactions between D98^Bid^ and G99^BHRF1^, and between R84^Bid^ and E89^BHRF1^.

The BHRF1–Puma BH3 complex showed a clear and continuous electron density map for BHRF1 residues 2–94 and 96–156, as well as Puma BH3 motif peptide residues 131–151 (Figure 2b). Similar to the BHRF1–Bid BH3 complex, BHRF1–Puma BH3 adopted a globular monomeric topology (Figure 1b). The four conserved hydrophobic residues of the Puma BH3 motif, I137, L141 M144, and L148, protrude into four hydrophobic pockets in the canonical ligand-binding groove, with an additional hydrophobic pocket occupied by a fifth hydrophobic residue W133 (Figure 2d). Furthermore, a conserved ionic interaction was also found in the BHRF1–Puma BH3 complex between R100^BHRF1^ and D146^Puma^. These salt bridges were supplemented by additional hydrogen bonds between the carboxyl group of E89^BHRF1^ and the NδH side-chain group of R135^Puma^, and between the G99^BHRF1^ main-chain amide group and the side-chain carboxyl and amide group of N149^Puma^ (Figure 2d).

**Figure 3:**
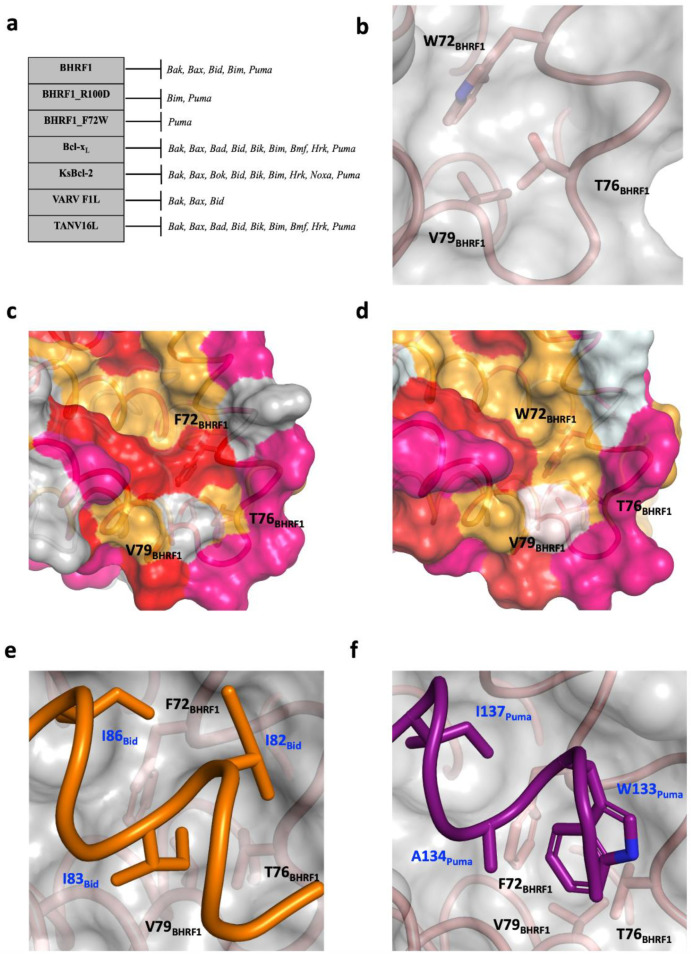
Interaction profiles and detailed view of the fifth hydrophobic pocket of BHRF1. (**a**) Summary of BH3 interaction profiles of BHRF1, BHRF1 R100D, and BHRF1 F72W mutant and its closely related Bcl-2 homologs, human Bcl-x_L,_ KSHV KsBcl-2, VARV F1L, and tanapoxvirus TANV16L. (**b**) Enlarged view of the additional hydrophobic pocket (fifth pocket) in the AlphaFold structure of F72W BHRF1 (**c**) View of the fifth pocket in BHRF1–BidBH3 with the ligand removed and colored according to residue hydrophobicity; selected residues of BHRF1 are labeled. (**d**) An identical view of F72W BHRF1 as in (**c**). The surface, hydrophobic pockets of the binding groove, and key residues are indicated. In (**c**,**d**), the surface is colored according to residue hydrophobicity: red: F, V, L, I, M; orange: W, Y, C, A, G, P, S, T; pink: N, E, H; gray: Q, R, K, D. (**e**) View of the fifth pocket in BHRF1–BidBH3. The surface of the BHRF1 is shown in gray with side-chains of the key hydrophobic residues located in the pocket shown as sticks. The BHRF1 backbone (salmon) and BidBH3 backbone (orange) are shown as tubes. Selected residues of Bid and BHRF1 are labeled. (**f**) Similar to (**e**), a view of the fifth hydrophobic pocket of BHRF1–Puma BH3 (purple).

In a search for BHRF1 variants with restricted ligand selectivity, Fitzsimmons et al. [20] investigated F72W BHRF1 and showed that it selectively bound Puma but much more weaky than the native sequence, and that it did not selectively bind Bid (Figure 3a). The lowered affinity of Puma is consistent with biological experiments showing that F72W BHRF1 does not protect cells from Puma-dependent apoptotic pathways [20]. In order to examine the possible molecular reasons for the alteration in the BH3 binding specificity for the F72W BHRF1 mutant, we generated an AlphaFold model [32]. The F72W BHRF1(1–160) AlphaFold model superimposition with the experimentally determined structure of BHRF1–PumaBH3 resulted in an RMSD value of 0.48 Å, confirming that the AlphaFold-generated structure was nearly identical to the experimental structures over the backbone atoms. Pocket 5 of F72W BHRF1 is shown in Figure 3b, and the main differences are small changes in the pocket including the presence of a hydrogen bond between T76 of the α3–α4 connecting loop and W72, in addition to a conformational change in the α3–α4 loop. A combination of altered steric and electrostatic interactions is likely responsible for the reduction in the ability of pocket 5 to accommodate a residue from the BH3 peptide. Figure 3b shows pocket 5 for F72W BHRF1, and Figure 3c,d show pocket 5 for F72W BHRF1 and BHRF1–BidBH3, respectively, colored by hydrophobicity. Figure 3e,f show the same region in the liganded forms of BHRF1 with BidBH3 and PumaBH3, respectively.

## 4. Discussion

**Figure 4:**
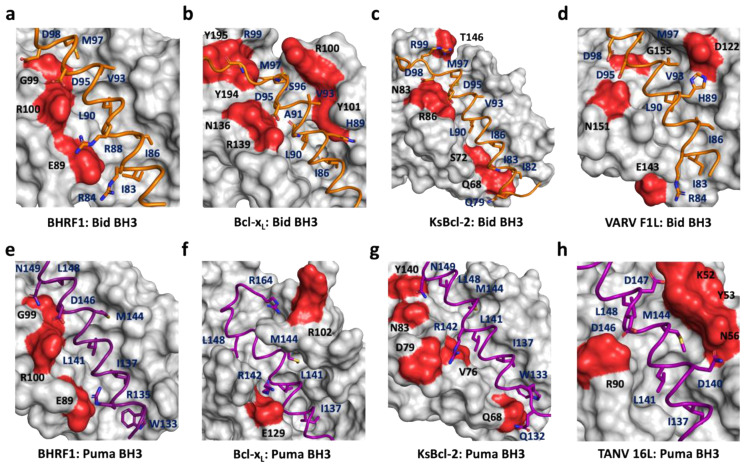
Comparison of BHRF1–Bid BH3 and Puma BH3 interaction sites with other closely related Bcl-2–BH3 complexes. (**a**) The BHRF1 surface and BH3 interaction sites are shown in gray and red respectively, whilst the Bid BH3 ribbon is shown in orange. (**b**) Human Bcl-x_L_–Bid BH3; (**c**) KSHV KsBcl-2–Bid BH3; (**d**) VARV F1L–Bid BH3; (**e**) BHRF1–Puma BH3; (**f**) human Bcl-x_L_–Puma BH3; (**g**) KSHV KsBcl-2–Puma BH3; (**h**) tanapoxvirus TANV16L–Puma BH3. The interaction formed by Bcl-2–BH3 residues and hydrophobic residues in the ligand-binding groove are labeled. Only residues located in the binding pockets are shown. Polar residues close to the BH3 ligand on the Bcl-2 protein are shown in red. The color schemes for the BH3 peptide and views in (**b**–**h**) are as in Figure 1.

Viruses have evolved countermeasures to hijack host-cell suicide to ensure cellular persistence and optimize viral replication [11,13,14]. Many viruses regulate intrinsic apoptosis through expressing homologs of the Bcl-2 family [11], including the oncogenic herpesvirus EBV. BHRF1, one of the two Bcl-2 homologs in the EBV genome, is known to stimulate tumorigenesis by inactivating the cellular proapoptotic proteins Bak, Bax, Bim, Bid, and Puma. Furthermore, this inhibition drives resistance to chemotherapy [18,20]. Here, we determined the crystal structures of EBV BHRF1 in complex with the BH3 motif peptides of human proapoptotic proteins Bid and Puma, two key BH3-only proteins in Bcl-2-regulated apoptosis. In contrast to Puma, Bid is activated by proteolytic cleavage by caspase-8 during death receptor-mediated apoptosis that results in a fragment, truncated Bid (tBid), bearing the BH3 motif that interacts with pro-survival Bcl-2 proteins [44]; however, like Bax and Bak, it can also elicit MOMP [45] and, therefore, links cell surface receptor-initiated apoptosis (extrinsic apoptosis) with intrinsic apoptosis. Bid interacts with five cellular pro-survival proteins (Bcl-2, Bcl-x_L_, Bcl-w, Mcl-1, and A1) to neutralize their activity but not with Bcl-B [46,47], and it additionally interacts with most viral Bcl-2 proteins with high affinity [23]. Although the primary protein sequence of BHRF1 significantly varies from its cellular Bcl-2 homologous counterparts (Table 2), it is highly conserved at a structural level (Figure 4). A detailed understanding of Bcl-2 family protein function in apoptosis regulation is not only crucial for identifying their biological function, but is important in the development of new therapeutic targets directed against this family [47,48,49]. Indeed, as a major mediator of programmed cell death, there is substantial interest in resolving the role of Bcl-2 family proteins at a molecular level with the aim of targeting them for their role in cancer progression [9,50].

Structural analysis of Bid and PumaBH3 motif interactions with BHRF1 presented here showed that binding occurred in a similar manner to what has previously been reported for viral and mammalian pro-survival Bcl-2–BH3 complexes [23]. A comparison of the BHRF1–Bid BH3 complex with KSHV KsBcl-2–Bid BH3 [17] or VARV F1L–Bid BH3 [51] shows that their mode of BH3 interaction is nearly identical, despite having significantly different affinities (110 nM, 20 nM, and 3220 nM toward Bid BH3, respectively). A similar trend can be observed in BHRF1–Puma BH3 and KsBcl-2–Puma BH3 [17] complexes, where BHRF1 shows nearly threefold reduced affinity for Puma BH3. Superimposition of the BHRF1 complex structures BHRF1–BidBH3 and BHRF1–Puma BH3 gave an RMSD value of 0.5 Å over the entire BHRF1 backbone, indicating the close structural similarity of the complexes. Pairwise analysis showed that the closest structural neighbors of the BHRF1–Bid and BHRF1–Puma complexes were KsBcl-2 from the herpesvirus KSHV, while the nearest mammalian homolog was human Bcl-x_L_. Although the Bcl-2 fold was highly similar (Figure 4), the BH3 binding selectivity differed (Figure 3a) [18].

A comparison between BHRF1 interactions with BidBH3 or PumaBH3 identified three conserved BHRF1 residues, E89, G99, and R100, in the binding groove which formed polar interactions with the BH3 motif peptides Bid or Puma (Figure 2 and Figure 4). Among these, E89 and R100 formed ionic interactions, and R100 made the highly conserved canonical salt bridge interaction with the conserved aspartic acid residue from the BH3 motif. This salt bridge is a hallmark interaction between pro-survival Bcl-2 protein and the BH3 motif of proapoptotic Bcl-2 protein [9], conserved across evolution [52,53], also reported for the BHRF1–BimBH3 and BHRF1–BakBH3 complexes [18]. The BH1 motif is located at the N-terminus of α5, and, although it generally conforms to the sequence XXGR, where XX are most commonly NW, but may vary, with the GR dipeptide very strongly conserved. In BHRF1, this BH1 region is SLGR, and the glycine and arginine residues make polar contacts with both Bid BH3 and Puma BH3 peptides. Site-directed mutagenesis studies in the BH1 motif of human pro-survival Bcl-2 proteins abolished BH3 binding [47], and similar behavior was reported with BHRF1. A charge reversal mutant in BHRF1 in the BH1 region, BHRF1 R100D, lost its affinity for Bid, Bak, and Bax and significantly lowered the affinity for BimBH3 (~15-fold reduction) and PumaBH3 (~6-fold reduction) [20]. These data suggest that residue R100 of BHRF1 is a crucial mediator for BH3 binding. Human Bcl-x_L_ and KSHV KsBcl2 have conserved BH1 NWGR motifs and show comparatively high affinities for both Bid and Puma [17,47]. In contrast, the structurally equivalent region to the NWGR motif in VARV F1L is replaced by the sequence LGVR, and the canonical salt bridge interaction is absent [51]. These observations highlight the importance of the BH1 equivalent region for determining interactions between Bcl-2 family proteins and provide a structural basis for the selectivity variation observed for proapoptotic BH3 ligands.

Structural analysis of the BHRF1 complexes showed the importance of a fifth hydrophobic pocket for BHRF1 BH3 ligand binding. Figure 4 presents a comparison of BidBH3 (Figure 4a–d) and Puma BH3 (Figure 4e–h) complexes. Examination of the BHRF1 and Bcl-x_L_ complexes liganded with Bid BH3 (Figure 4a,b) revealed that Bid BH3 utilized the canonical four conserved hydrophobic residues of the BH3 motif peptides to interact with four hydrophobic pockets of the Bcl-2 binding groove. In contrast to Bcl-x_L_–Bid BH3 (Figure 4b) and the previously determined structures of BHRF1–Bim and Bak BH3 motif complexes [18], Bid BH3 engages a fifth hydrophobic pocket of BHRF1 (Figure 3 and Figure 4a). Residue I83^Bid^ occupies a pocket formed by BHRF1 residues F72, T76, and V79 (Figure 3e), all residues highly conserved or conservatively substituted in BHRF1 variants. The use of a fifth hydrophobic pocket has previously been observed in several other viral Bcl-2–Bid BH3 complexes such as KsBcl-2 (Figure 4c) and the poxvirus variola virus F1L (Figure 4d) [17,51]. The fifth hydrophobic pocket of BHRF1 is utilized by Puma BH3, where the structurally equivalent residue to Bid I83 is A134 or W133 (Figure 3f and Figure 4e). The binding mode for Bcl-x_L_–Puma BH3 (Figure 4f) differs from that of the BHRF1 complex. The fifth pocket has previously been observed in the KSHV KsBcl-2 Puma complex (Figure 4g) and tanapox 16L, showing the variation found within the exact interactions that the viral Bcl-2 proteins can form in binding the same ligand (Figure 4h). Binding studies have emphasized the importance of the fifth pocket to BHRF1 to ligand selectivity and affinity. Fitzsimmons et al. showed that F72W BHRF1 lost binding to almost all BH3 peptides except for PumaBH3, which showed a ~4-fold reduction in affinity (Figure 3a) [20]. The predicted changes rendered in pocket 5 by the presence of the Trp residue include changes in backbone α3–α4 loop conformation, as well as an altered conformation of Thr76, and these facets change the hydrophobicity of the pocket (Figure 3c,d). Collectively, these changes have the effect of disfavoring Bid binding but only reducing Puma BH3 affinity (Figure 3a). An alternative fifth hydrophobic pocket has been observed for the orfvirus Bcl-2 protein Puma complex, ORFV125–Puma BH3, using residue Y152^Puma^ [54], as well as for Bax BH3 complexes where the residue Bax BH3 M74 uses a fifth pocket in complexes with Mcl-1, Bcl-x_L_ [55], Bcl-2 [56], ORFV125 [57], and SPPV14 [36]. Collectively, these data suggest that virus-encoded pro-survival Bcl-2 proteins make extensive use of an additional hydrophobic pocket to bind select host pro-death Bcl-2 proteins, which has implications for selectivity and affinity for host pro-death Bcl-2 proteins by their viral Bcl-2 antagonists. Conversely, obstruction of one of the four canonical hydrophobic pockets is employed by the ancient pro-survival Bcl-2 protein BHP2 from a sponge [58]. Clearly, the flexibility within the Bcl-2 fold enables multiple avenues to achieve BH3 interactor selectivity. Intriguingly, the most promiscuous host pro-death Bcl-2 protein Bim has not been shown to use a fifth hydrophobic pocket whilst typically displaying high affinities, but it can be bound with high selectivity by several virus-encoded Bcl-2 proteins including vaccinia virus F1L [59] and grouper iridovirus GIV66 [60], as well as by human Bcl-b [46].

Apoptosis induction by viral infection has been shown to be dependent on Puma for apoptosis via Bax/Bak activation [61]; viral infection leads to caspase-8 activation and, hence, Bid cleavage and activation [14]. Thus, inhibiting both Puma and Bid can play significant roles in suppressing virus induced apoptosis. Understanding the precise atomic details of the BHRF1 Puma and Bid complexes will potentially allow us to target the interactions in a molecular design process. The exploitation of the fifth hydrophobic pocket of BHRF1 presents a unique opportunity for designing specific inhibitors. Inhibition of BHRF1 has been shown to induce apoptosis in EBV-infected cells [62], holding out the prospect that small-molecule inhibitors of BHRF1 may be of utility in inducing apoptosis in EBV-infected cells. Our findings may provide important targets for the design of peptidomimetic drugs to mimic these interactions, and the structures we present here provide the mechanistic basis that could be potentially exploited for targeting EBV infections.

## Figures and Tables

**Table 1:** X-ray crystallographic data collection and refinement statistics. Values in parentheses are for the highest resolution shell.

	BHRF1–Bid BH3 (PDB ID 7P33)	BHRF1–Puma BH3 (PDB ID 7P9W)
**Data collection**		
Space group	P6_5_22	P3_2_21
Cell dimensions		
a, b, c (Å)	94.20, 94.20, 455.58	62.77, 62.77, 92.60
α, β, γ (°)	90, 90, 120	90, 90,120
Wavelength (Å)	0.9537	0.9537
Resolution (Å)	46.7–2.78 (2.88–2.78)	35.25–2.00 (2.07–2.0)
R_sym_ or R_merge_	0.04 (0.38)	0.05 (0.51)
I/σI	7.86 (1.14)	6.76 (1.15)
Completeness (%)	98.8 (90.9)	99.7 (99.9)
CC1/2	0.99 (0.66)	0.99 (0.57)
Redundancy	2.0 (2.0)	2.0 (2.0)
**Refinement**		
Resolution (Å)	46.7–2.78 (2.88–2.78)	35.25–2.00 (2.07–2.0)
No. reflections	32,688	14,737
Rwork/Rfree	0.231/0.274	0.217/0.252
Clashscore	1.20	5.18
No. atoms		
Protein	7192	1422
Ligand/ion	9	57
Water	62	54
B-factors		
Protein	62.68	42.03
Ligand/ion	104.78	51.63
Water	51.29	49.48
R.m.s. deviations		
Bond lengths (Å)	0.002	0.005
Bond angles (°)	0.40	0.73

**Table 2:** Percentage sequence identity of BHRF1 with its mammalian counterparts.

	Bcl-2	Bcl-x_L_	Bcl-w	Mcl-1	Bfl-1/A1	Bcl-B
BHRF1	21.7%	17.9%	18.4%	9.6%	17.8	21.5%

## Data Availability

Data supporting the findings of this manuscript are available from the corresponding authors upon reasonable request. Coordinate files were deposited at the Protein Data Bank (https://www.rcsb.org/, accessed on 13 March 2021) using accession codes 7P33 and 7P9W for the BHRF1–BidBH3 and BHRF1–PumaBH3 complexes, respectively. The raw X-ray diffraction data were deposited at the SBGrid Data Bank [33] (https://data.sbgrid.org/data/, accessed on 1 January 2020) using their PDB accession codes.
